# Graphene Oxide Modulates ROS Production and Apoptotic Responses to Bortezomib in Human Glioblastoma Cells: An In Vitro Study

**DOI:** 10.3390/cells15151417

**Published:** 2026-08-05

**Authors:** Rafał Krętowski, Agata Jabłońska-Trypuć, Natalia Tyszka, Joanna Kalita, Marzanna Cechowska-Pasko

**Affiliations:** 1 Department of Pharmaceutical Biochemistry, Medical University of Bialystok, Mickiewicza 2A, 15-222 Bialystok, Poland; rafal.kretowski@umb.edu.pl (R.K.); natalia.tyszka@umb.edu.pl (N.T.); 2 Department of Chemistry, Biology and Biotechnology, Bialystok University of Technology, Wiejska 45A, 15-351 Bialystok, Poland; a.jablonska@pb.edu.pl; 3 Department of Biology and Pharmacognosy, Medical University of Bialystok, Mickiewicza 2A, 15-222 Bialystok, Poland; joanna.kalita@umb.edu.pl

**Keywords:** apoptosis, cytotoxicity, glioblastoma, oxidative stress, bortezomib, graphene oxide

## Abstract

Glioblastoma multiforme (GBM) remains one of the most aggressive and treatment-resistant brain tumors, characterized by rapid proliferation and poor patient prognosis. Novel therapeutic strategies are urgently needed to improve clinical outcomes. In this study, we investigated the cytotoxic and pro-apoptotic effects of bortezomib (BORT), a proteasome inhibitor, and graphene oxide (GO), a nanomaterial with known anticancer potential, on human glioblastoma cell lines. Treatment with BORT and GO, both individually and in combination, significantly reduced cell viability in a dose-dependent manner, as determined by MTT. In this study, we observed enhanced apoptotic cell death, accompanied by increased activation of both caspase-8 and caspase-9, indicating simultaneous engagement of extrinsic and intrinsic apoptotic pathways. Western blot analysis demonstrated downregulation of anti-apoptotic proteins Bcl-2 and upregulation of pro-apoptotic markers (NOXA, cleaved PARP). A central finding of this work is the pronounced increase in intracellular reactive oxygen species (ROS) levels following BORT–GO treatment. The elevated ROS levels observed in BORT–GO-treated cells compared with free bortezomib therefore suggest that GO-mediated oxidative stress may amplify proteasome inhibition-induced apoptosis, which is particularly visible in the A172 and LN229 cell lines. Notably, the combination of BORT and GO may suggest a potential cooperative mechanism through proteasome inhibition and oxidative stress induction. These findings indicate that graphene oxide may modulate the antitumor efficacy of bortezomib in a cell line-dependent manner and support further investigation of this combination as a promising therapeutic approach for glioblastoma.

## 1. Introduction

Glioblastoma (GBM) is the most common and aggressive primary malignant brain tumor in adults and continues to be associated with a dismal prognosis. Despite multimodal treatment consisting of maximal surgical resection followed by radiotherapy and temozolomide-based chemotherapy, median overall survival rarely exceeds 15 months. The limited therapeutic efficacy is primarily associated with pronounced intratumoral heterogeneity, intrinsic and acquired resistance to treatment, and the persistence of glioblastoma stem-like cells, which contribute to tumor recurrence and therapeutic failure. Consequently, the identification of new therapeutic approaches capable of overcoming resistance mechanisms and promoting efficient tumor cell elimination remains a major clinical challenge.

Targeting the ubiquitin–proteasome system has emerged as a promising strategy for cancer therapy because of its central role in maintaining protein homeostasis and regulating multiple signaling pathways involved in cell survival. Bortezomib (BORT), the first clinically approved reversible inhibitor of the 26S proteasome, has demonstrated substantial therapeutic benefit in multiple myeloma and several other hematological malignancies. Although its clinical application in solid tumors is still under investigation, growing evidence indicates that bortezomib may also exert antitumor activity against GBM. Inhibition of proteasomal degradation results in the accumulation of misfolded and pro-apoptotic proteins, induction of endoplasmic reticulum stress, and disruption of cellular proteostasis, ultimately leading to apoptotic cell death. In glioblastoma models, bortezomib has been reported to enhance apoptosis through several mechanisms, including NOXA upregulation, NF-κB inhibition, and Mcl-1 cleavage, while also sensitizing differentiated glioblastoma cells and glioma stem-like cells to death receptor-mediated apoptosis, including TRAIL-dependent signaling pathways [1]. In glioma models, bortezomib also exhibits synergism with temozolomide and can suppress tumor growth via modulation of the FOXM1–Survivin axis [2]. However, efficacy in GBM is limited by compensatory survival programs, protective autophagy responses, and incomplete penetration of the blood–brain barrier.

Parallel to small-molecule strategies, nanomaterials have emerged as versatile tools in cancer therapy, capable of drug delivery, photothermal therapy, and direct cytotoxic effects. Among them, graphene oxide (GO) has attracted considerable interest due to its high surface area, biocompatibility, and ease of functionalization. In a variety of cancer models, GO has been shown to inhibit tumor cell proliferation, induce oxidative stress, and trigger apoptosis, often through mitochondrial pathways or modulation of signaling cascades [3]. In glioblastoma, graphene-based nanomaterials have demonstrated the capacity to reduce tumor volume, suppress invasiveness, and induce apoptotic cell death [4]. Mechanistically, GO may induce ROS generation, mitochondrial dysfunction, and downstream caspase activation, as well as regulate autophagy pathways [5]. Moreover, GO’s surface can be engineered to carry therapeutic cargo, further enhancing its utility in anti-cancer drug regimens [6]. Recent studies have demonstrated that graphene oxide-based nanoplatforms can improve the delivery and bioavailability of anticancer agents, thereby enhancing therapeutic efficacy. At the same time, accumulating evidence indicates that the biological effects of graphene-based nanomaterials are highly dependent on their physicochemical properties—including size, surface chemistry, oxidation state, and functionalization—and the molecular characteristics of the target cells, emphasizing the need for context-dependent evaluation of their therapeutic potential [7,8,9].

Despite the individual promise of bortezomib and graphene oxide in cancer therapy, their combined effects in glioblastoma remain largely unexplored. Although graphene oxide has been extensively investigated as a nanocarrier for anticancer compounds, little is known about its interaction with proteasome inhibitors in glioblastoma cells. The present study was designed to evaluate the anticancer activity of the proteasome inhibitor bortezomib, both as a single agent and in combination with graphene oxide, in four human glioblastoma cell lines. This investigation focused on determining its impact on cell growth inhibition and apoptotic cell death. Using established in vitro GBM models, we assessed multiple cellular responses, including changes in cell viability, proteasome function, apoptosis induction, expression of apoptosis- and endoplasmic reticulum stress-related markers, activation of key apoptotic signaling proteins, and alterations in intracellular reactive oxygen species (ROS) levels.

## 2. Materials and Methods

### 2.1. Reagents

Cell culture reagents: Dulbecco’s Modified Eagle Medium (DMEM) supplemented with high glucose (4.5 mg/mL) and GlutaMax™, fetal bovine serum Gold (FBS Gold), phosphate-buffered saline (PBS), trypsin-EDTA, penicillin, and streptomycin were purchased from Gibco (San Diego, CA, USA). The MTT reagent [3-(4,5-dimethylthiazol-2-yl)-2,5-diphenyltetrazolium bromide] was obtained from Merck (St. Louis, MO, USA). Bortezomib was supplied by MedChemExpress (Monmouth Junction, NJ, USA). RIPA lysis buffer and Protease/Phosphatase Inhibitor Cocktail were purchased from Cell Signaling Technology (Danvers, MA, USA).

Flow cytometry reagents: Cell apoptosis and mitochondrial membrane potential were evaluated using the FITC Annexin V Apoptosis Detection Kit I and the JC-1 MitoScreen Kit (BD Pharmingen™, San Diego, CA, USA), respectively. Caspase-8 and caspase-9 activities were determined using the FAM-LETD-FMK Caspase-8 Assay and the FAM-LEHD-FMK Caspase-9 Assay, whereas intracellular reactive oxygen species (ROS) levels were measured with the Intracellular Total ROS Activity Assay (ImmunoChemistry Technologies, Davis, CA, USA).

Western blot reagents: The following primary antibodies were obtained from Cell Signaling Technology (Danvers, MA, USA): phospho-eIF2α (Ser51) (D9G8), CHOP (L63F7), BCL-2 (D17C4), NOXA (D8L7U), PARP (P09874), and β-tubulin. Horseradish peroxidase (HRP)-conjugated anti-rabbit IgG secondary antibody and enhanced chemiluminescence (ECL) detection reagent were also purchased from the same supplier. Polyvinylidene difluoride (PVDF) membranes for protein transfer were obtained from Bio-Rad Laboratories (Hercules, CA, USA). The Pierce™ ECL Western Blotting Substrate and the Pierce™ BCA Protein Assay Kit were purchased from Thermo Fisher Scientific (Rockford, IL, USA).

Additional reagents: Graphene oxide was provided by AGP (Zielona Góra, Poland). The fluorogenic proteasome substrate N-Suc-LLVY-AMC and bovine serum albumin (BSA) were obtained from Sigma-Aldrich (St. Louis, MO, USA). Bio-Rad Protein Assay Dye Reagent Concentrate was purchased from Bio-Rad Laboratories (Hercules, CA, USA).

### 2.2. Physicochemical Characteristics of GO

Graphene oxide (GO) was obtained from Advanced Graphene Products (AGP, Zielona Góra, Poland) as a 1 wt% aqueous dispersion (product code XBB/GO/1%, flake size < 20 µm). According to the manufacturer, the material consists of single- to few-layer graphene oxide sheets produced by chemical oxidation and exfoliation of graphite by the method of Hummers. GO is characterized by the presence of numerous oxygen-containing functional groups, including hydroxyl, epoxy, carbonyl, and carboxyl moieties. These surface functionalities confer high hydrophilicity, promote excellent dispersibility, and ensure good colloidal stability in aqueous solutions. The material used in this study had a maximum lateral flake size of less than 20 μm. According to previous reports describing graphene oxide synthesized using similar oxidation protocols, GO typically consists of approximately 11–20 stacked layers with an individual sheet thickness ranging from 0.7 to 0.9 nm, corresponding to single- or few-layer graphene oxide. Such materials generally exhibit a carbon-to-oxygen atomic ratio between 2.0 and 3.5 and a negative surface charge, with a zeta potential of approximately −30 to −50 mV in aqueous dispersions. Owing to its large specific surface area together with the high abundance of oxygen-containing functional groups, graphene oxide demonstrates strong adsorption capacity, high water dispersibility, and enhanced ability to interact with a wide range of biological molecules. These physicochemical properties make GO suitable for biomedical and toxicological applications.

### 2.3. Cell Culture and Exposure to Bortezomib (BORT), Graphene Oxide (GO) and Combination Treatment (MIX: BORT + GO)

Human glioblastoma cell lines A172, LN229, T98G, and U118-MG were purchased from the American Type Culture Collection (ATCC, Manassas, VA, USA). Cells were maintained in Dulbecco’s Modified Eagle Medium (DMEM) supplemented with 10% heat-inactivated fetal bovine serum Gold (FBS Gold), 100 U/mL penicillin, and 100 μg/mL streptomycin, following the supplier’s recommended culture conditions. Cell cultures were incubated at 37 °C in a humidified atmosphere containing 5% CO_2_ using a Galaxy S+ incubator (RS Biotech, Irvine, UK). Dulbecco’s phosphate-buffered saline (DPBS; pH 7.4) was used for routine washing procedures.

For each experiment, approximately 2.5 × 10^5^ cells were seeded in 2 mL of complete culture medium per well of six-well plates. After reaching the desired level of confluence, cells were detached with 0.05% trypsin containing 0.02% EDTA, and cell numbers were determined using a Scepter Cell Counter (Millipore, Burlington, MA, USA). The required number of cells was subsequently plated for individual assays. Following a 24 h attachment period, the culture medium was replaced with fresh medium containing either bortezomib (BORT) at cell line-specific IC_50_ concentrations (A172, 3.5 nM; LN229, 33 nM; T98G, 7 nM; U118-MG, 10 nM), graphene oxide (100 μg/mL), or a combination of both agents (MIX: BORT + GO). The selected GO concentration (100 µg/mL) based on previous literature reports and preliminary dose–response observations obtained in our experimental system. Following treatment, the cells were maintained for an additional 48 h under standard culture conditions (37 °C, humidified atmosphere containing 5% CO_2_) before being collected for subsequent analyses. Untreated cultures, maintained in the absence of GO served as the negative control group. Throughout this study, cells were used at passage numbers not exceeding 10. Prior to the experiments, the identity of all cell lines was verified by short tandem repeat (STR) profiling. In addition, cultures were routinely screened for mycoplasma contamination using a PCR-based detection assay, and all tested samples remained mycoplasma-free during the entire experimental period.

### 2.4. Cell Viability Assay

The effect of bortezomib on glioblastoma cell viability was evaluated using the MTT assay [10]. Human GBM cell lines (A172, LN229, T98G, and U118-MG) were seeded into 24-well plates at a density of 0.5 × 10^5^ cells per well and allowed to attach before treatment. Cells were then exposed to increasing concentrations of bortezomib (5–400 nM) for either 24 or 48 h.

Following incubation, the culture medium was removed, and cells were washed three times with phosphate-buffered saline (PBS). Subsequently, 1 mL of MTT solution (0.25 mg/mL in PBS) was added to each well, and the plates were incubated for 1 h at 37 °C in a humidified atmosphere containing 5% CO_2_. After removal of the MTT solution, the resulting formazan crystals were dissolved in 0.5 mL of 0.1 mol/L HCl prepared in absolute isopropanol. Absorbance was determined at 570 nm using a microplate reader (Tecan, Männedorf, Switzerland). Cell viability was expressed as a percentage relative to untreated control cells. Each experiment was carried out in triplicate and independently repeated using three separate cell cultures.

### 2.5. Proteasome Activity Assay

Proteasome activity was determined in whole-cell lysates prepared by sonication in lysis buffer containing 50 mM Tris-HCl (pH 7.5), 150 mM NaCl, 1 mM EDTA, 1 mM EGTA, and 0.5% Triton X. Following cell disruption, lysates were centrifuged at 12,000× *g* for 15 min at 4 °C to remove cellular debris. Protein concentration in the resulting supernatants was quantified using the Bio-Rad Protein Assay Dye Reagent Concentrate (Bio-Rad Laboratories, Hercules, CA, USA) according to the manufacturer’s instructions, with bovine serum albumin (BSA) used for generation of the standard curve. Absorbance was measured using a BioPhotometer (Eppendorf, Hamburg, Germany).

For the enzymatic assay, aliquots containing 20 μg of total protein were transferred into 96-well microplates (Corning Inc., Corning, NY, USA). Samples were incubated in proteasome assay buffer (100 mM Tris-HCl, pH 7.5, 1 mM EDTA, and 1 mM EGTA) supplemented with 100 μmol/L of the fluorogenic substrate succinyl-Leu-Leu-Val-Tyr-7-amido-4-methylcoumarin (Suc-LLVY-AMC; Sigma-Aldrich, St. Louis, MO, USA), as previously described [11]. Fluorescence was monitored for 30 min at 37 °C using a FLUOStar OPTIMA microplate reader (BMG Labtech GmbH, Offenburg, Germany), with measurements recorded every 2 min (excitation: 355 nm; emission: 460 nm).

Proteasome activity was expressed as the amount of 7-amino-4-methylcoumarin (AMC) released from the substrate per minute (pmol/min) and normalized to the total protein content (U/mg). Each experimental condition was analyzed in triplicate.

### 2.6. Detection of Apoptosis and Necrosis

The proportions of apoptotic and necrotic cells were determined by flow cytometry using an FACSCanto II flow cytometer (BD Biosciences, San Diego, CA, USA). A172, LN229, T98G, and U118-MG cells were seeded into six-well plates at a density of 2.0 × 10^5^ cells per well in 2 mL of complete culture medium. After a 24 h attachment period, the medium was replaced with fresh medium containing either bortezomib (BORT) at the respective IC_50_ values (A172, 3.5 nM; LN229, 33 nM; T98G, 7 nM; U118-MG, 10 nM), graphene oxide (100 μg/mL), or the combined treatment (MIX: BORT + GO). Cells were then incubated for 48 h under standard culture conditions (37 °C, 5% CO_2_).

Following treatment, cells were harvested, pelleted by centrifugation at 1000 rpm for 5 min at room temperature, and washed with Dulbecco’s phosphate-buffered saline (DPBS, pH 7.4). The cell pellet was resuspended in binding buffer and stained with fluorescein isothiocyanate (FITC)-conjugated Annexin V and propidium iodide (PI) using the FITC Annexin V Apoptosis Detection Kit I (BD Pharmingen™, San Diego, CA, USA). Staining was carried out for 15 min at room temperature in the absence of light. Samples were analyzed using FACSDiva software Version 6.1.3 (BD Biosciences, San Diego, CA, USA). All data were obtained from three independent experiments.

### 2.7. Measurement of Caspase-8 and Caspase-9 Activity

The enzymatic activities of caspase-8 and caspase-9 were determined using the FAM-LETD-FMK Caspase-8 Assay and the FAM-LEHD-FMK Caspase-9 Assay (ImmunoChemistry Technologies, Davis, CA, USA). A172, LN229, T98G, and U118-MG cells were exposed to bortezomib (BORT), graphene oxide (GO), or their combination (MIX: BORT + GO) for 48 h prior to analysis. Caspase activation was assessed according to the manufacturer’s protocol, following the procedure previously described in our earlier studies [12,13].

### 2.8. Determination of Total Protein Concentration

The total protein content of cell lysates was quantified using the Pierce™ BCA Protein Assay Kit (Thermo Fisher Scientific, Rockford, IL, USA) in accordance with the manufacturer’s instructions [14]. A standard calibration curve was prepared using bovine serum albumin (BSA) at concentrations of 25, 50, 100, 150, 200, 300, and 400 μg/mL. Protein concentrations in the analyzed samples were subsequently calculated from the corresponding standard curve.

### 2.9. Western Blot Analysis

Following the indicated treatments, A172, LN229, T98G, and U118-MG cells were rinsed with ice-cold phosphate-buffered saline (PBS; pH 7.4) and lysed in 300 μL of RIPA buffer supplemented with a protease and phosphatase inhibitor cocktail. Cell lysates were clarified by centrifugation at 10,000× *g* for 15 min at 4 °C, after which the protein concentration of the supernatants was determined.

Equal amounts of protein (15 μg per sample) were separated on 12% SDS-polyacrylamide gels according to the method of Laemmli [15] using a constant electrophoretic current of 25 mA. Following electrophoresis, proteins were transferred onto polyvinylidene difluoride (PVDF) membranes. To minimize nonspecific antibody binding, membranes were blocked for 1 h at room temperature in Tris-buffered saline containing 0.05% Tween 20 (TBS-T) supplemented with 5% non-fat dry milk.

After three washes with TBS-T, membranes were incubated overnight at 4 °C with the appropriate primary antibodies diluted 1:1000 in TBS containing 5% non-fat dry milk. Excess primary antibody was removed by washing with TBS-T before incubation with horseradish peroxidase (HRP)-conjugated anti-rabbit IgG secondary antibody (1:1000 dilution in TBS) for 2 h at room temperature. Following additional washing steps, immunoreactive protein bands were visualized using an enhanced chemiluminescence (ECL) detection reagent.

### 2.10. Chemiluminescence Detection

Immunoreactive protein bands were visualized using a luminol-based enhanced chemiluminescence (ECL) substrate specific for horseradish peroxidase (HRP). Images were acquired with the GeneGnome chemiluminescence imaging system (Syngene, Frederick, MD, USA), and densitometric analysis of the detected bands was performed using GeneTools software version 4.3.8.0 (Syngene, Frederick, MD, USA).

### 2.11. Measurement of Intracellular Reactive Oxygen Species (ROS)

Intracellular total reactive oxygen species (ROS) levels were quantified using the Intracellular Total ROS Activity Assay (ImmunoChemistry Technologies, Davis, CA, USA). Fluorescence intensity was analyzed by flow cytometry with a FACSCanto II cytometer (BD Biosciences, San Diego, CA, USA) using the standard FITC detection channel (530/30 nm).

A172, LN229, T98G, and U118-MG cells were seeded at a density of 2.0 × 10^5^ cells per well in DMEM and allowed to attach for 24 h. The culture medium was then replaced with fresh medium containing bortezomib (BORT), graphene oxide (GO), or the combined treatment (MIX: BORT + GO), followed by incubation for an additional 48 h.

After treatment, cells were harvested, adjusted to a concentration of 1 × 10^6^ cells/mL, and collected by centrifugation at 200× *g*. The resulting cell pellet was resuspended in 1× Assay Buffer, and 10 μL of Total ROS Green reagent was added to 490 μL of each cell suspension. Samples were incubated for 1 h at 37 °C in a humidified atmosphere containing 5% CO_2_ before flow cytometry analysis.

### 2.12. Statistical Analysis

All results are presented as the mean ± standard deviation (SD) of three independent experiments. Statistical analyses were performed using GraphPad Prism version 9 (GraphPad Software, San Diego, CA, USA). The choice of statistical test was based on the distribution of the data. Comparisons among multiple groups were carried out using one-way analysis of variance (ANOVA) followed by Tukey’s post hoc test for normally distributed data, whereas non-parametric data were analyzed using the Kruskal–Wallis test followed by Dunn’s multiple comparisons post hoc test.

## 3. Results

### 3.1. Effect of Bortezomib on Glioblastoma Cell Viability

**Figure 1:**
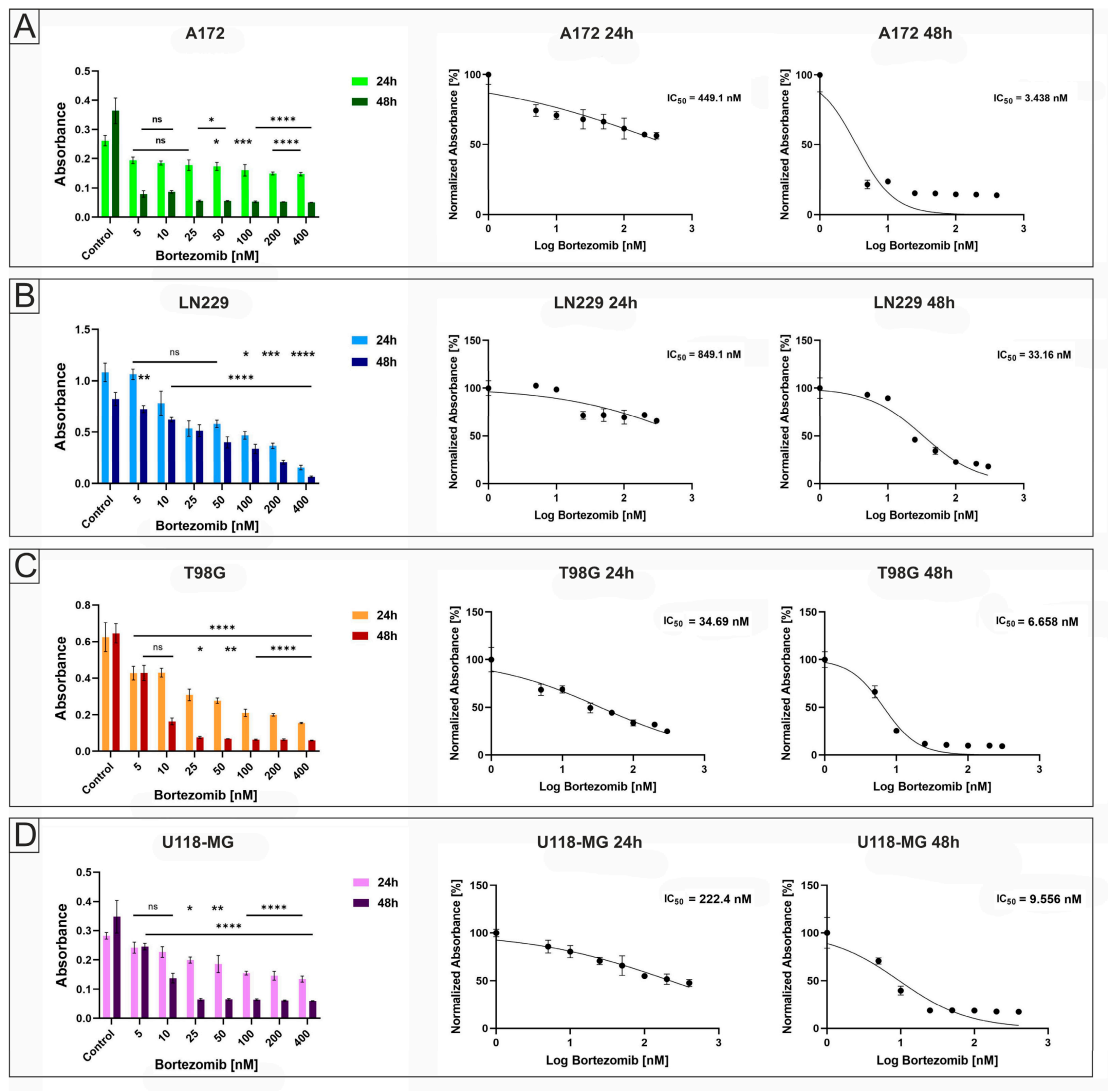
Effect of bortezomib on the viability of human glioblastoma cell lines A172 (**A**), LN229 (**B**), T98G (**C**), and U118-MG (**D**). Cells were exposed to increasing concentrations of bortezomib (5–400 nM) for 24 or 48 h, after which cell viability was determined using the MTT assay. The bar graphs (**left panels**) present cell viability at each treatment time, expressed relative to untreated control cells, whereas the corresponding dose–response curves (**right panels**) were used to determine the IC_50_ values. The results are shown as the mean ± SD of three independent experiments, each performed in triplicate. Statistical significance is indicated as follows: * *p* < 0.05, ** *p* < 0.01, *** *p* < 0.001, **** *p* < 0.0001; ns, not significant (*p* > 0.05). Statistical analyses were selected according to the distribution of the data and included one-way ANOVA followed by Tukey’s post hoc test or the Kruskal–Wallis test followed by Dunn’s multiple comparisons post hoc test, as appropriate.

The cytotoxic effect of bortezomib on glioblastoma cells is summarized in Figure 1. Cell viability was evaluated in four human glioblastoma cell lines (A172, LN229, T98G, and U118-MG) following exposure to bortezomib at concentrations ranging from 5 to 400 nM. The MTT assay was performed after 24 and 48 h of treatment to determine the time- and concentration-dependent effects of the compound on cell viability. The results are shown both as bar graphs (absorbance values) and dose–response curves with calculated IC_50_ values.

Bortezomib treatment significantly impaired the viability of all four glioblastoma cell lines examined (A172, LN229, T98G, and U118-MG). The magnitude of this effect increased with increasing drug concentration and prolonged treatment duration (Figure 1A–D). All cell lines exhibited markedly increased sensitivity after 48 h compared with 24 h of treatment. A172 and LN229 cells were relatively resistant at 24 h (IC_50_ = 449.1 nM and 849.1 nM, respectively), but showed a pronounced shift toward nanomolar sensitivity at 48 h (IC_50_ = 3.438 nM and 33.16 nM). T98G cells were highly sensitive already at 24 h (IC_50_ = 34.69 nM), with further enhancement after 48 h (IC_50_ = 6.658 nM). U118-MG cells displayed intermediate sensitivity at 24 h (IC_50_ = 222.4 nM), which increased substantially following prolonged exposure (IC_50_ = 9.556 nM). These findings demonstrate a strong time-dependent increase in bortezomib cytotoxicity across glioblastoma cell lines.

For all four glioblastoma cell lines, bortezomib reduces cell viability in a dose-dependent manner. The cytotoxic effect becomes much stronger after 48 h, reflected in considerably lower IC_50_ values. Among the tested lines, A172 and T98G appear most sensitive to bortezomib, especially at 48 h. These results suggest that prolonged exposure enhances the drug’s anticancer activity across different glioblastoma models.

### 3.2. Effect of Bortezomib and Graphene Oxide on the Activity of Proteasomes in Glioblastoma Cells

**Figure 2:**
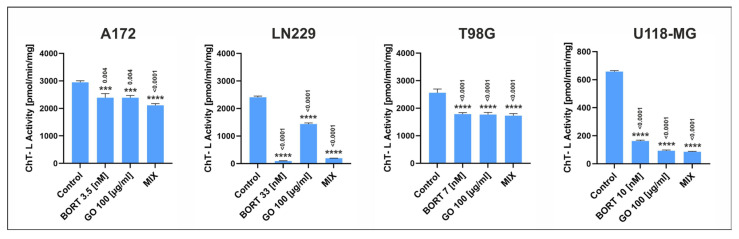
Effect of bortezomib (BORT), graphene oxide (GO, 100 µg/mL), combination treatment (MIX: BORT + GO) and control (untreated) after 48 h on the chymotrypsin-like activity of proteasome [pmol/min/mg of protein] in four glioblastoma cell lines: A172, LN229, T98G and U118-MG. Data are presented as the mean ± standard deviation (SD) of three independent experiments, each performed in triplicate. Statistical significance is indicated as follows: *** *p* < 0.004, **** *p* < 0.0001. Depending on data distribution, comparisons between groups were performed using one-way analysis of variance (ANOVA) followed by Tukey’s post hoc test.

Figure 2 presents the chymotrypsin-like activity of proteasome in four glioblastoma cell lines: A172, LN229, T98G, and U118-MG following treatment with: bortezomib (BORT), graphene oxide (GO, 100 µg/mL), combination treatment (MIX: BORT + GO) and control (untreated) after 48 h. Bortezomib significantly reduced the chymotrypsin-like activity of proteasome in all treated glioblastoma cell lines A172, LN229, T98G, and U118-MG (Figure 2).

### 3.3. Effect of Bortezomib and Graphene Oxide on Apoptosis and Necrosis

**Figure 3:**
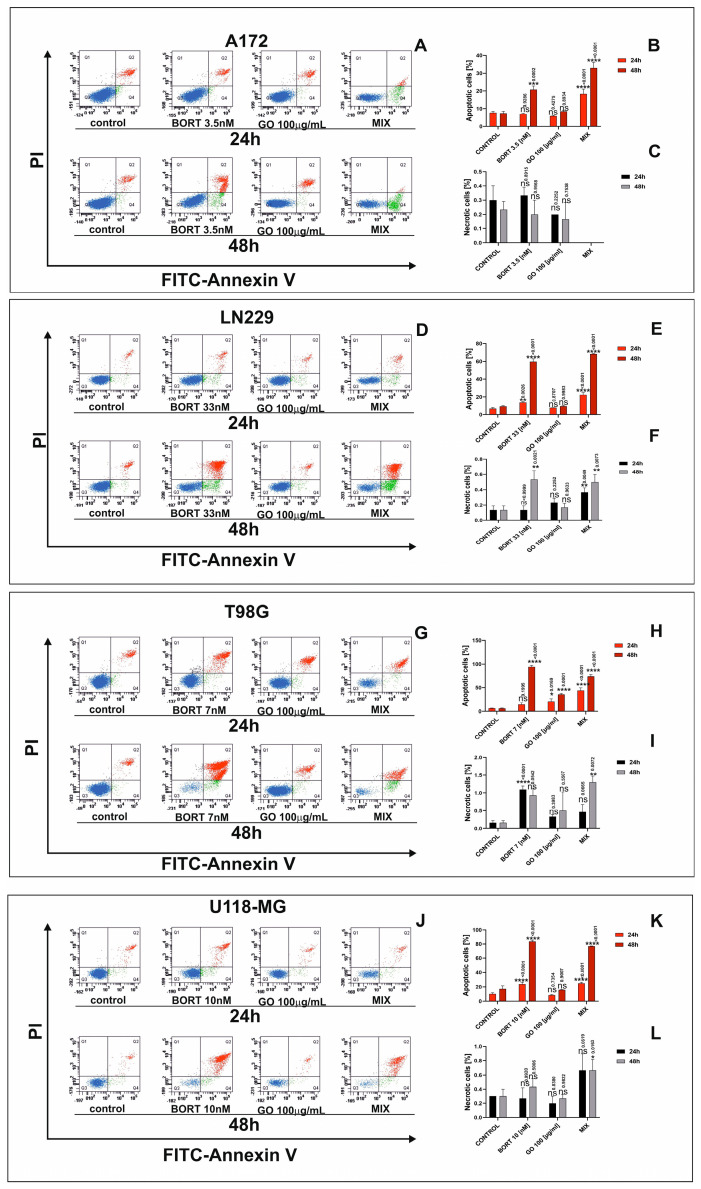
Effect of bortezomib (BORT), graphene oxide (GO; 100 μg/mL), and their combination (MIX: BORT + GO) on apoptosis and necrosis in A172, LN229, T98G, and U118-MG glioblastoma cells after 24 and 48 h of treatment. Representative Annexin V/propidium iodide (PI) flow cytometry dot plots are shown for A172 (**A**–**C**), LN229 (**D**–**F**), T98G (**G**–**I**), and U118-MG (**J**–**L**) cells. Viable cells (Annexin V^−^/PI^−^) are located in quadrant Q3, early apoptotic cells (Annexin V^+^/PI^−^) in Q4, late apoptotic cells (Annexin V^+^/PI^+^) in Q2, and necrotic cells (Annexin V^−^/PI^+^) in Q1. Quantitative analysis is presented in the bar graphs. The percentage of apoptotic cells was calculated as the sum of early and late apoptotic populations (Q4 + Q2) and is shown in panels (**B**,**E**,**H**,**K**), whereas the percentage of necrotic cells is presented in panels (**C**,**F**,**I**,**L**). Data are presented as the mean ± standard deviation (SD) of three independent experiments, each performed in triplicate. Statistical significance is indicated by asterisks, ns—not statistically significant. Depending on data distribution, comparisons between groups were performed using one-way analysis of variance (ANOVA) followed by Tukey’s post hoc test.

Flow cytometry analysis of apoptosis and necrosis in A172 (Figure 3A–C), LN229 (Figure 3D–F), T98G (Figure 3G–I), and U118-MG (Figure 3J–L) glioblastoma cells following treatment with bortezomib (BORT), graphene oxide (GO; 100 μg/mL), their combination (MIX: BORT + GO), or vehicle control is presented in Figure 3. Cells were analyzed after 24 and 48 h using Annexin V-FITC/propidium iodide (PI) staining. In the flow cytometry analysis, viable cells were identified as Annexin V^−^/PI^−^ (Q3), early apoptotic cells as Annexin V^+^/PI^−^ (Q4), late apoptotic cells as Annexin V^+^/PI^+^ (Q2), and necrotic cells as Annexin V^−^/PI^+^ (Q1). The percentage of apoptotic cells was calculated as the combined proportion of early and late apoptotic populations (Q2 + Q4), whereas necrotic cells were quantified separately.

Compared with untreated controls, all investigated glioblastoma cell lines demonstrated a reduction in the proportion of viable cells accompanied by an increase in apoptotic cell populations following treatment. However, the magnitude of this response varied among the different cell lines. Exposure to bortezomib alone significantly promoted apoptosis in A172 (Figure 3A,B), LN229 (Figure 3D,E), T98G (Figure 3G,H), and U118-MG (Figure 3J,K) cells, with the most pronounced effect observed after 48 h of treatment. In contrast, graphene oxide alone exerted only a limited pro-apoptotic effect, which reached significance only in the T98G cell line (Figure 3H).

Combined treatment with graphene oxide and bortezomib (MIX: BORT + GO) resulted in a further increase in apoptosis in the A172 (Figure 3A,B) and LN229 (Figure 3D,E) cell lines compared with bortezomib treatment alone. In contrast, the combination treatment caused a weaker apoptotic response than bortezomib alone in the T98G (Figure 3G,H) and U118-MG (Figure 3J,K) cell lines.

Necrosis was significantly increased following bortezomib treatment in the LN229 (Figure 3F) and T98G (Figure 3I) cell lines. The combination of graphene oxide and bortezomib modestly increased the percentage of necrotic cells in the LN229 (Figure 3F), T98G (Figure 3I), and U118-MG (Figure 3L) cell lines.

### 3.4. Effect of Bortezomib and Graphene Oxide on ER Stress in Glioblastoma Cells

**Figure 4:**
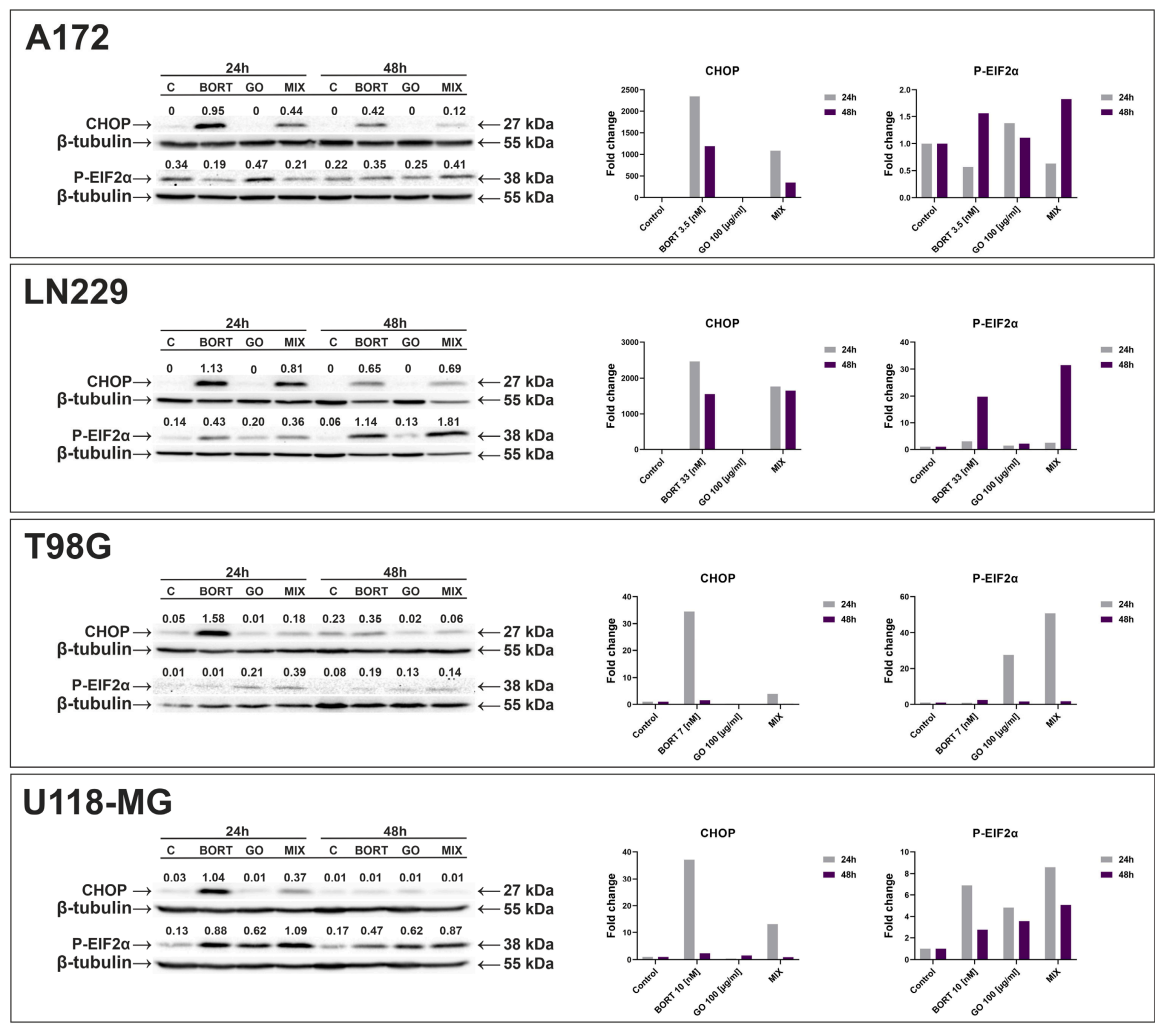
Representative Western blot analysis of CHOP and phosphorylated eIF2α (P-eIF2α) expression in A172, LN229, T98G, and U118-MG glioblastoma cell lines following treatment with bortezomib (BORT), graphene oxide (GO; 100 μg/mL), or their combination (MIX: BORT + GO) for 24 and 48 h. Equal amounts of protein (15 μg per sample) were separated by SDS-PAGE and analyzed by immunoblotting. Representative immunoblots for CHOP, P-eIF2α, and β-tubulin are shown. β-Tubulin (55 kDa) served as the loading control. Band intensities were quantified by densitometric analysis, normalized to β-tubulin, and the corresponding normalized values are indicated above each band. Protein expression is presented as the fold change relative to untreated control cells (C), for which the normalized expression level was defined as 1.

To investigate whether the changes occurring in bortezomib and graphene oxide-treated cells were induced by ER stress, we performed a Western blot analysis to determine the expression of CHOP and P-EIF2α proteins (Figure 4) in four glioblastoma cell lines: A172, LN229, T98G, and U118-MG.

These results showed an increase in the expression of both ER-stress-related proteins, particularly after 24 h of bortezomib and MIX: BORT + GO treatment, in all tested cell lines. The expression of CHOP protein (Figure 4) significantly decreases after 48 h for all treatments. The acquired data suggest that the bortezomib and MIX: BORT + GO may affect ER homeostasis and induce ER stress in the tested cell lines.

### 3.5. Effect of Bortezomib and Graphene Oxide on the Expression of Apoptosis Markers in Glioblastoma Cells

**Figure 5:**
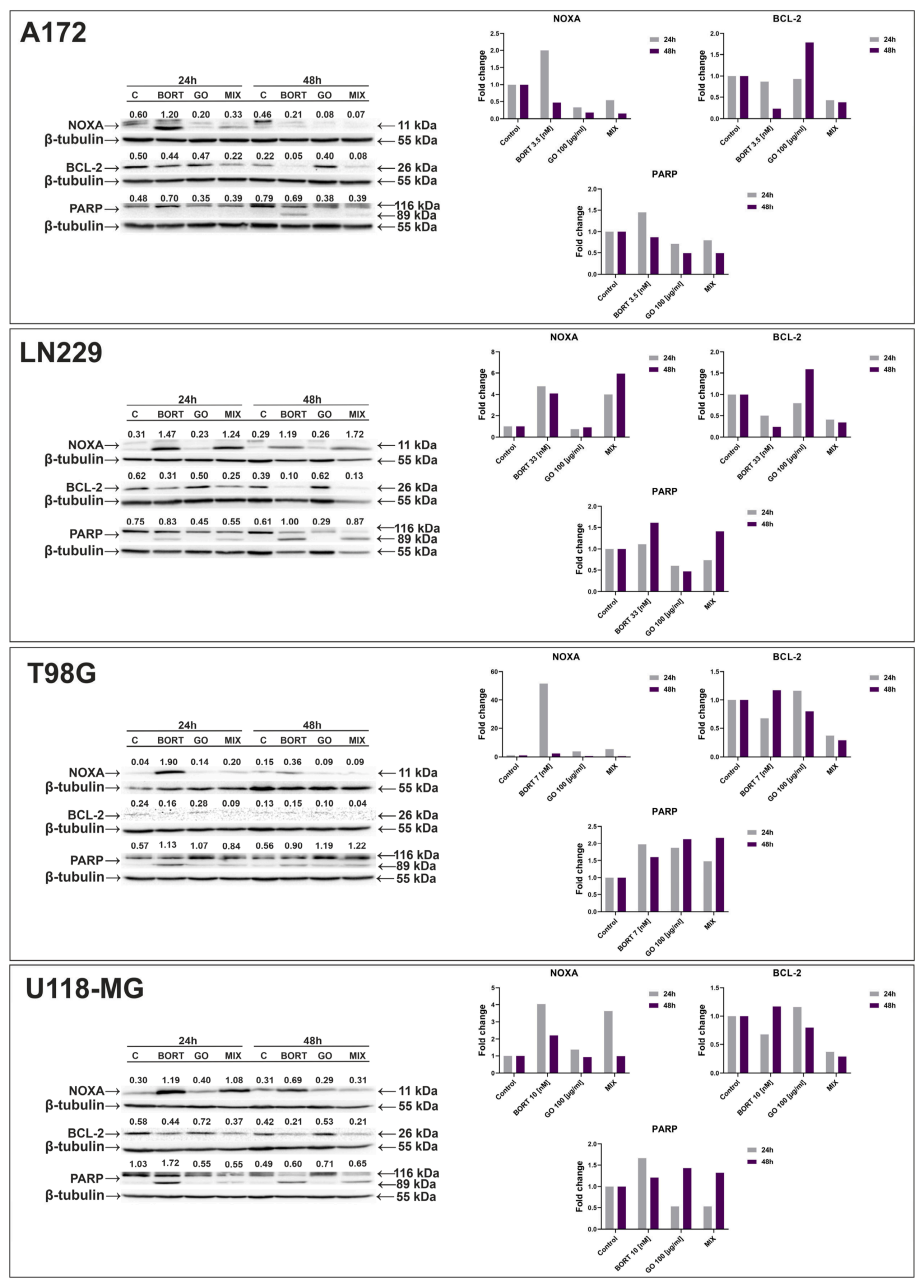
Representative Western blot analysis of NOXA, BCL-2, and cleaved PARP protein expression in A172, LN229, T98G, and U118-MG glioblastoma cell lines following treatment with bortezomib (BORT), graphene oxide (GO; 100 μg/mL), or their combination (MIX: BORT + GO) for 24 and 48 h. Equal amounts of protein (15 μg per sample) were resolved by SDS-PAGE and subjected to immunoblot analysis. Representative immunoblots of NOXA, BCL-2, cleaved PARP, and β-tubulin are shown. β-Tubulin (55 kDa) was used as the loading control for normalization. Protein band intensities were quantified by densitometry, normalized to β-tubulin, and the resulting normalized values are indicated above the corresponding bands. Relative protein expression is presented as fold change compared with untreated control cells (C), for which the normalized expression level was assigned a value of 1.

To determine the effect of bortezomib (BORT), graphene oxide (100 µg/mL) and combination treatment (MIX: BORT + GO) on the apoptosis of the researched glioblastoma cell lines, an analysis of apoptosis marker levels was made. We performed a Western blot analysis to determine the expression of pro-apoptotic proteins NOXA, cleaved PARP and the anti-apoptotic protein BCL-2 (Figure 5) in four: A172, LN229, T98G, and U118-MG glioblastoma cell lines. Furthermore, densitometric analysis and calculations for the expressions of NOXA, cleaved PARP and BCL-2 proteins were performed and compared to a stable loading control (β-tubulin) for correcting for unequal loading (left side of Figure 5). Then, these normalized values are compared to a control group (e.g., untreated cells) to determine fold changes (right side of Figure 5).

The obtained results indicate an increase effect of bortezomib and graphene oxide on NOXA and cleaved PARP proteins expression in LN229, T98G, and U118-MG (Figure 5) compared to the control, untreated cells. However, the expressions of anti-apoptotic protein BCL-2 under the influence of bortezomib and graphene oxide decreased in all tested cell lines, compared to the control cells (Figure 5).

### 3.6. Effect of Bortezomib and Graphene Oxide on Caspase-8 and Caspase-9 Activation

**Figure 6:**
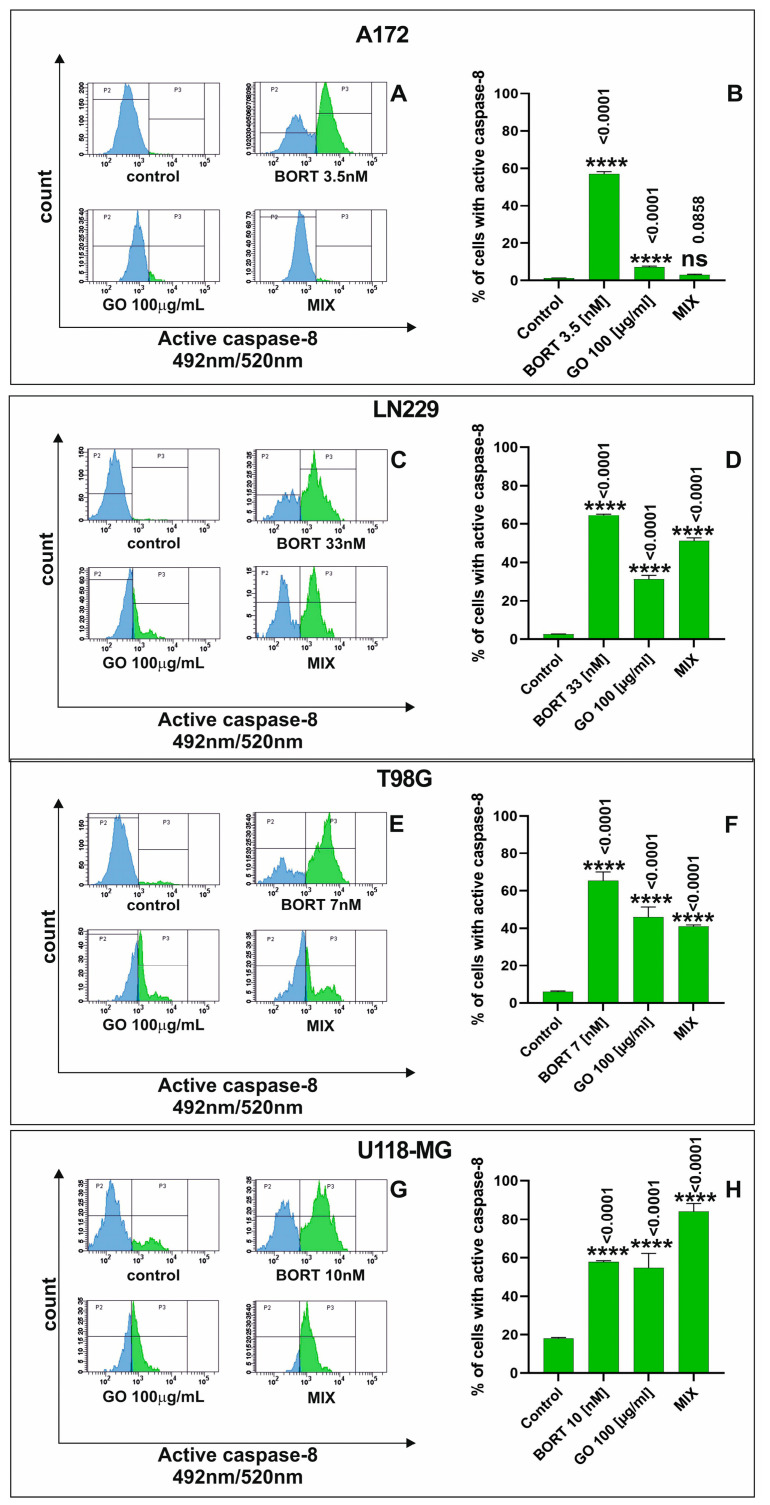
Effect of bortezomib (BORT), graphene oxide (GO; 100 μg/mL), and their combined treatment (MIX: BORT + GO) on caspase-8 activation in glioblastoma cell lines after 48 h of exposure. Flow cytometry analysis of active caspase-8 is presented for A172 (**A**,**B**), LN229 (**C**,**D**), T98G (**E**,**F**), and U118-MG (**G**,**H**) cells. Representative fluorescence histograms obtained using the FAM-LETD-FMK Caspase-8 Assay are shown in the left panels (**A**,**C**,**E**,**G**), while the corresponding quantitative analysis of the percentage of caspase-8-positive cells is presented in the right panels (**B**,**D**,**F**,**H**). Gate P2 represents the population of cells without detectable caspase-8 activation, whereas gate P3 corresponds to cells exhibiting active caspase-8. Data are presented as the mean ± standard deviation (SD) of three independent experiments, each performed in triplicate. Statistical significance is indicated by asterisks, ns—not statistically significant. Depending on data distribution, comparisons between groups were performed using one-way analysis of variance (ANOVA) followed by Tukey’s post hoc test.

**Figure 7:**
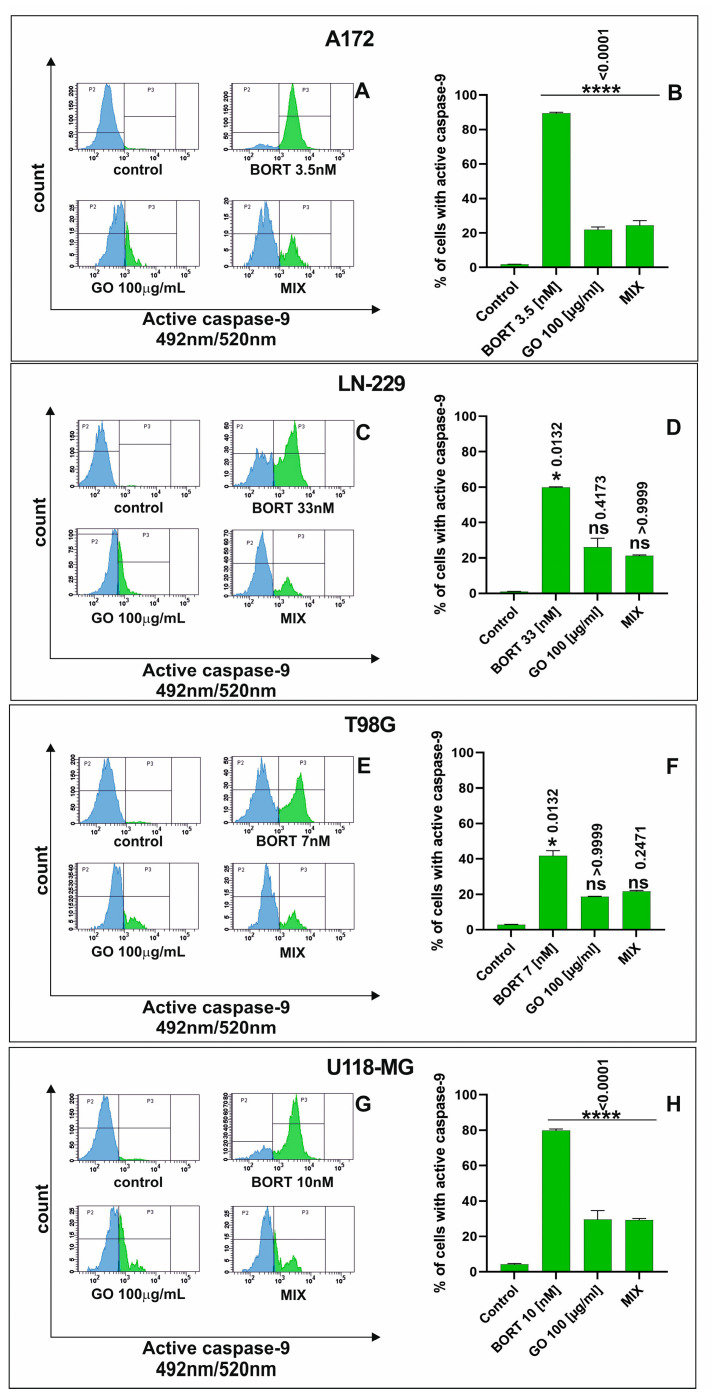
Effect of bortezomib (BORT), graphene oxide (GO; 100 μg/mL), and their combination (MIX: BORT + GO) on caspase-9 activation in A172, LN229, T98G, and U118-MG glioblastoma cell lines following 48 h of treatment. Flow cytometry analysis of active caspase-9 is shown for A172 (**A**,**B**), LN229 (**C**,**D**), T98G (**E**,**F**), and U118-MG (**G**,**H**) cells. Representative fluorescence histograms obtained with the FAM-LEHD-FMK Caspase-9 Assay are presented in the left panels (**A**,**C**,**E**,**G**), whereas the corresponding quantitative analysis of the percentage of cells with activated caspase-9 is shown in the right panels (**B**,**D**,**F**,**H**). Gate P2 indicates the population lacking detectable caspase-9 activity, while gate P3 represents cells exhibiting active caspase-9. Data are shown as the mean ± SD. Statistical significance is denoted by asterisks, ns—not statistically significant. Group comparisons were performed using one-way ANOVA with Tukey’s post hoc test or, for non-normally distributed data, the Kruskal–Wallis test followed by Dunn’s post hoc test.

In the present study, the potential effects of 48 h of incubation with bortezomib (BORT), graphene oxide (GO, 100 µg/mL) and combination treatment (MIX: BORT + GO) on the activation of caspase-8 (Figure 6) and caspase-9 (Figure 7) were investigated.

The results of this study demonstrate cell line-specific responses. We observed an increase in % of cells with active caspase-8, after exposure to all treatment, compared to control cells the with exception of combination treatment in A172. Moreover, our data presented an increase in % of cells with active caspase-9, after exposure to all treatment, compared to control cells with the exception of GO and combination treatment in LN229 and T98G cells. It is worth noting that in A172 cells, the addition of GO strongly attenuated the activation of caspase-8 and caspase-9 induced by bortezomib alone, in contrast to U118-MG cells, where we observed a strong increase in caspase-8 activation in combination treatment.

### 3.7. Effect of Bortezomib and Graphene Oxide on ROS Production in Glioblastoma Cells

**Figure 8:**
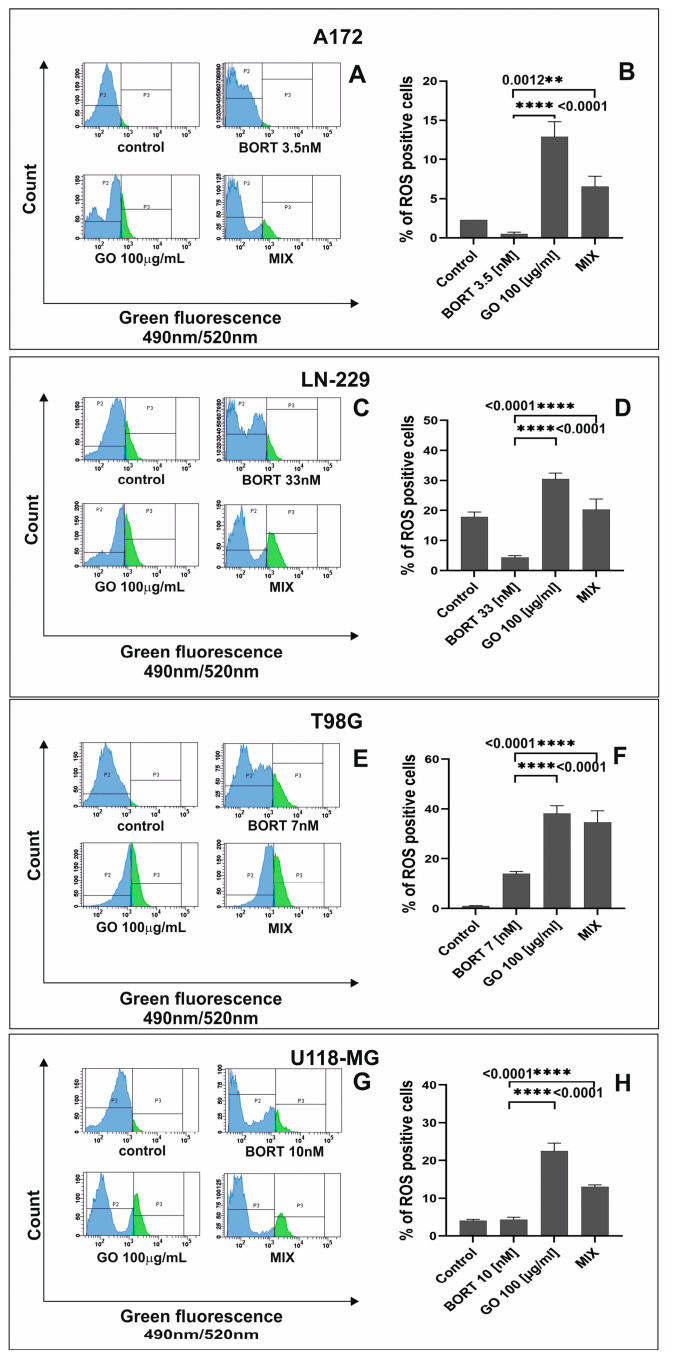
The influence of bortezomib (BORT), graphene oxide (GO, 100 µg/mL) and combination treatment (MIX: BORT + GO) on ROS production in A172 (**A**,**B**), LN229 (**C**,**D**), T98G (**E**,**F**), and U118-MG (**G**,**H**) cell lines for 48 h. Gate P2—negative ROS cells, gate P3—positive ROS cells. Mean values ± SD from three independent experiments performed in triplicate are presented. Significant alterations are marked with asterisks. Data are presented as the mean ± standard deviation (SD) of three independent experiments, each performed in triplicate. Statistical significance is indicated as follows: ** *p* = 0.0012, **** *p* < 0.0001. Depending on data distribution, comparisons between groups were performed using one-way analysis of variance (ANOVA) followed by Tukey’s post hoc test.

Figure 8 shows the effects of bortezomib (BORT), graphene oxide (GO, 100 µg/mL) and combination treatment (MIX: BORT + GO) on intracellular ROS synthesis in the tested glioblastoma cell lines for 48 h. Intracellular total ROS production was evaluated by flow cytometry following fluorescent labeling with the Intracellular Total ROS Activity Assay kit. Incubating A172, LN229, T98G and U118-MG glioblastoma cells with bortezomib alone we did not observe induction of ROS production in these cells compared to control cells. It is very interesting that all treated cell lines displayed the significant induction of ROS synthesis with 100 µg/mL of GO and with combination treatment (MIX: BORT + GO) in comparison to bortezomib alone.

## 4. Discussion

Bortezomib is a first-in-class proteasome inhibitor whose anticancer activity has been extensively linked to induction of apoptosis through disruption of protein degradation and accumulation of pro-apoptotic factors [16,17,18]. Despite its clinical success, particularly in hematological malignancies, resistance and dose-limiting toxicity significantly restrict its broader application [19,20]. Consequently, nanomaterial-based delivery systems have been widely investigated as a strategy to enhance the intracellular efficacy of bortezomib while mitigating its limitations [21,22].

In this study, we evaluated the cytotoxic effects of the proteasome inhibitor bortezomib alone and in combination with graphene oxide across four human glioblastoma cell lines. Our results demonstrate a robust dose- and time-dependent reduction in cell viability following bortezomib treatment, accompanied by decreased proteasome activity, induction of apoptosis, modulation of ER stress markers, activation of key apoptotic proteins, and variable effects on ROS production. These findings expand the understanding of proteasome targeting in GBM and highlight nuanced responses to nanomaterial adjuncts such as graphene oxide.

Bortezomib significantly reduced viability in all tested glioblastoma cell lines in a time-dependent manner, with markedly lower IC_50_ values after 48 h compared to 24 h. All cell lines show dramatically lower IC_50_ values at 48 h vs. 24 h, indicating proteotoxic stress accumulates over time, misfolded proteins overwhelm the cells’ capacity to adapt, and apoptotic pathways are likely activated after prolonged exposure. This suggests that sustained proteasome inhibition is crucial for maximal therapeutic effect.

This pattern aligns with literature showing that proteasome inhibition has an anti-proliferative effect in GBM cells by disrupting protein turnover and cell-cycle regulation, as evident in preclinical glioma models and other solid tumors treated with bortezomib or related inhibitors. Proteasome inhibitors disturb homeostatic degradation of cell cycle and survival proteins, leading to accumulation of pro-apoptotic factors and inhibition of survival pathways (e.g., NF-κB, survivin) in various malignancies including glioma and pancreatic carcinoma models [23]. The pronounced reduction in cell viability in our dataset supports this established mechanism and confirms the sensitivity of A172 and T98G lines in particular.

Differences in sensitivity of lines may correlate with known genetic backgrounds. A172 cells are often sensitive to proteasome inhibition, likely due to their high metabolic demand, potentially driven by aberrant EGFR signaling commonly observed in glioblastoma. This can lead to increased accumulation of misfolded or unfolded proteins and elevated proteotoxic stress [24,25]. Upon proteasome inhibition with bortezomib, this excessive proteotoxic burden cannot be resolved, triggering unfolded protein response (UPR)-mediated apoptosis and rendering the cells more sensitive to treatment [26].

LN229 cells, despite harboring mutant p53 similar to T98G, exhibit greater resistance to bortezomib. This suggests that LN229 cells may activate alternative survival pathways, particularly PI3K/AKT signaling and autophagy, which are frequently upregulated in glioblastoma and can compensate for impaired proteasome function [27,28]. These mechanisms may allow LN229 cells to better tolerate bortezomib-induced proteotoxic stress.

Although T98G cells are typically resistant to temozolomide (TMZ) due to high MGMT expression, they demonstrate increased sensitivity to bortezomib. Proteasome inhibition has been shown to suppress MGMT expression, at least partially via inhibition of NF-κB signaling, thereby sensitizing glioblastoma cells to therapy [25,29]. In this context, bortezomib functionally overcomes MGMT-mediated resistance.

The genetic profile of U118-MG places it among more aggressive glioblastoma models, characterized by PTEN loss and constitutive activation of the PI3K/AKT pathway [30]. Although this signaling axis promotes survival and therapeutic resistance, the mesenchymal phenotype of U118-MG is associated with increased secretory activity and metabolic demand, which may impose a high proteostatic burden [31]. Consequently, proteasome inhibition may induce a rapid and lethal proteotoxic crisis in these cells, similar to that observed in highly secretory tumor types [25].

Consistent with the reduction in viability, bortezomib markedly inhibited chymotrypsin-like proteasome activity across all cell lines, confirming target engagement and mechanistic specificity. Proteasome inhibition in glioma cells has been associated with apoptosis induction through accumulation of misfolded proteins, blockade of cell-cycle progression, and disruption of degradation of pro-apoptotic factors (e.g., p21, p27) [23]. Our functional proteasome data corroborate the central role of the ubiquitin–proteasome system in GBM cell survival and highlight that bortezomib’s cytotoxicity is likely mediated by this primary mechanism.

Flow cytometry analysis revealed strong significant increases in apoptotic cell fractions with bortezomib treatment in all cell lines. However, the results of this study demonstrate cell line-specific responses. It is important that combined treatment with graphene oxide and bortezomib (MIX: BORT + GO) resulted in a further increase in apoptosis in the A172 and LN229 cell lines compared with bortezomib treatment alone. Notably, bortezomib induced stronger apoptosis than GO or combination treatments in the T98G and U118-MG cell lines, indicating that proteasome inhibition is the dominant pro-apoptotic driver in this context. This observation is in line with previous research indicating heterogeneity in apoptosis sensitivity across glioma models and suggests intrinsic or acquired resistance mechanisms may modulate cell death response.

Necrotic cell fractions increased slightly only in LN229 and T98G cell lines after bortezomib treatment and in combined treatment with botrezomib in LN229, T98G and U118-MG cell lines. Such mixed death modalities have been observed in other cancers treated with proteasome inhibitors, which can trigger both apoptotic and non-apoptotic pathways when proteostatic stress is overwhelming [32,33]. The lack of effect of GO alone on necrosis and apoptosis suggests that at the concentration tested (100 µg/mL), GO does not substantially augment cell death.

Our Western blot analysis demonstrated increased expression of ER stress markers CHOP and P-EIF2α particularly at 24 h after bortezomib and MIX treatments. This is consistent with proteasome inhibition causing accumulation of misfolded proteins in the ER, triggering the unfolded protein response (UPR) and subsequent stress pathways that favor apoptosis when sustained. Prior studies in GBM and other cancer types have linked proteasome inhibitor-induced ER stress to apoptotic signaling and enhanced caspase activation, even in solid tumors where bortezomib has limited clinical efficacy [34]. The transient nature of CHOP elevation at 24 h with reduction by 48 h may reflect adaptive cellular responses or progression toward terminal apoptosis. The short-term increase in CHOP and phosphorylated EIF2α observed after 24 h suggests activation of the unfolded protein response during the early phase of treatment. Nevertheless, additional intermediate time points together with pharmacological modulation of ER stress would be required to determine whether ER stress represents an upstream regulator of apoptosis or a secondary consequence of proteasome inhibition. Additional functional experiments employing ER stress inhibitors or modulators would further clarify whether ER stress is a causative mechanism or a secondary adaptive response as an important direction for future studies. 

Our data reveal increased expression of pro-apoptotic protein NOXA, decreased levels of anti-apoptotic BCL-2, and activation of initiator caspases (caspase-8 and caspase-9) after bortezomib exposure. In the present study, BORT–GO treatment resulted in marked activation of caspase-8 in the case of the LN229, T98G and U118-MG lines, indicating engagement of the extrinsic apoptotic pathway. Previous reports have demonstrated that bortezomib can sensitize cancer cells to death receptor-mediated apoptosis by suppressing NF-κB signaling and upregulating death receptors such as DR4 and DR5 [17,35,36]. The enhanced caspase-8 activation in combined treatment with bortezomib and GO in U118-MG cells suggests that graphene oxide may modulate these mechanisms, consistent with studies showing that GO improves intracellular drug delivery and membrane interaction in cancer cells [37,38].

In parallel, significant activation of caspase-9 was observed, indicating involvement of the intrinsic, mitochondria-dependent apoptotic pathway. Proteasome inhibition has been shown to induce mitochondrial dysfunction, cytochrome c release, and apoptosome formation, ultimately leading to caspase-9 activation [17,39,40]. Importantly, simultaneous activation of caspase-8 and caspase-9 suggests functional crosstalk between extrinsic and intrinsic pathways, which has been reported to amplify apoptotic signaling and overcome apoptosis resistance in cancer cells [41,42]. These molecular events are hallmarks of intrinsic and extrinsic apoptosis pathways and align with literature showing that proteasome inhibition can upregulate NOXA while suppressing survival pathways to trigger mitochondrial membrane permeabilization and caspase cascade activation.

A central finding of this work is the pronounced increase in intracellular ROS levels following GO and BORT–GO treatment. Oxidative stress is a well-recognized contributor to bortezomib-induced cytotoxicity, as excessive ROS disrupt mitochondrial membrane potential and promote intrinsic apoptosis [43,44]. Graphene oxide has independently been reported to induce ROS generation through mitochondrial perturbation and redox imbalance, particularly in cancer cells [45,46,47]. The elevated ROS levels observed in GO and BORT–GO in all treated cell lines compared with free bortezomib in which GO-mediated oxidative stress amplifies proteasome inhibition-induced apoptosis is interpreted as cooperative or context-dependent effect. The future studies employing formal synergy analyses, such as Combination Index (Chou–Talalay), Bliss independence, or Loewe additivity models, will be necessary to quantitatively determine the nature of the interaction.

Notably, ROS has also been shown to enhance death receptor signaling and caspase-8 activation, providing a mechanistic link between oxidative stress and extrinsic apoptosis [48]. This interaction may explain the coordinated activation of both apoptotic pathways observed in this study. Similar multi-pathway apoptotic responses have been reported for other nanocarrier-based anticancer systems, supporting the concept that nanomaterials can function as active modulators of intracellular signaling rather than passive drug carriers [49,50].

Contrary to some reports in other cancer models where proteasome inhibition elevates ROS and contributes to apoptosis, we did not detect significant ROS induction with bortezomib alone. This discrepancy may reflect cell line-specific redox regulation or effective antioxidant defenses in GBM cells. Notably, GO and the MIX induced ROS in all tested cell lines, suggesting that oxidative stress might play a context-dependent role. This effect may reflect differences in mitochondrial engagement and/or intrinsic antioxidant capacity of the studied glioblastoma cells. Regarding the functional role of ROS in apoptosis induction, that experiments using ROS scavengers such as N-acetylcysteine (NAC) would provide valuable mechanistic insight into whether oxidative stress acts as a driver or a downstream consequence of cell death. NAC-based functional assays combined with apoptosis analyses (e.g., Annexin V staining and caspase activation) as an important direction for future studies. Literature indicates that ROS generation in response to proteasome inhibition can vary between models, with some cancers relying on ROS for apoptosis induction while others do not exhibit robust oxidative changes [34]. These findings imply that ROS may not be a consistent mediator of bortezomib cytotoxicity in GBM, but could be influenced by additional stressors or combination strategies.

Moreover, the receptor-mediated signaling pathways are potentially involved in glioblastoma progression and therapy resistance. In particular, known is the role of angiotensin II/AGTR1 signaling, which has been associated with enhanced tumor cell proliferation, migration, angiogenesis, and resistance to apoptosis in glioblastoma. Emerging evidence indicates that pharmacological inhibition of this pathway may exert anti-tumor effects by modulating inflammatory signaling, oxidative stress, and tumor microenvironment interactions. Panza et al. [51] demonstrated that losartan-mediated blockade of AGTR1 signaling suppresses glioblastoma cell viability and interferes with oncogenic downstream pathways. These observations support the concept that receptor-mediated pathways, including AGTR1-associated signaling networks, may represent relevant therapeutic targets and could contribute to the biological interpretation of the results.

Although proteasome inhibitors such as bortezomib demonstrate potent in vitro activity against GBM cells, clinical translation has been hindered by limited brain penetration and modest efficacy in solid tumors, compared with hematologic malignancies where these drugs are approved therapies [23]. Our findings reinforce the value of proteasome targeting in GBM research but also highlight the complexity of cellular responses, the importance of apoptosis and ER stress pathways, and the potential need for combination strategies that enhance drug delivery or overcome resistance mechanisms.

While graphene oxide has been proposed as a nanocarrier to enhance drug delivery and anticancer activity, our results indicate only moderate effects when applied alone or in combination with bortezomib at the selected concentration. This suggests that GO’s intrinsic cytotoxic or chemo-sensitizing properties may be limited under these conditions or require optimization of physicochemical properties, dosage, or delivery mechanisms for enhanced synergy, as seen in other preclinical GBM models [33].

Although our study demonstrates that combined GO and bortezomib treatment alters apoptosis and oxidative stress, it does not directly address the physicochemical interaction between both components. Since graphene oxide possesses a high adsorption capacity for numerous therapeutic compounds, future studies should determine bortezomib loading efficiency, adsorption kinetics, and release characteristics. Such analyses will clarify whether GO primarily acts as a nanocarrier facilitating intracellular drug delivery, as an independent inducer of oxidative stress, or through both mechanisms simultaneously.

An important observation of the present study is the heterogeneous response among different glioblastoma cell lines. Whereas GO enhanced bortezomib-induced apoptosis in A172 and LN229 cells, this effect was less pronounced in T98G and U118-MG cells. Such differences most likely reflect intrinsic molecular heterogeneity of glioblastoma, including variability in proteasome activity, antioxidant capacity, apoptotic signaling, and stress-response pathways. Further mechanistic studies will be required to identify the determinants responsible for these distinct cellular responses.

The current work was performed exclusively using conventional two-dimensional cultures, which facilitate mechanistic analyses but do not fully reproduce the complex glioblastoma microenvironment. Future validation in three-dimensional spheroids, patient-derived organoids, and in vivo models will be essential to assess the translational relevance of the observed effects.

## 5. Conclusions

Overall, bortezomib exhibits significant anti-glioblastoma activity through dose- and time-dependent cytotoxicity, proteasome inhibition, apoptosis induction, and ER stress engagement. The variable ROS responses and limited additive effect of GO underscore the importance of dissecting cell line-specific mechanisms. Collectively, these findings are consistent with accumulating evidence that graphene oxide-based nanoplatforms can enhance the anticancer efficacy of chemotherapeutic agents by increasing intracellular drug accumulation, promoting oxidative stress, and simultaneously activating multiple apoptotic pathways. Such multi-level apoptotic induction is particularly relevant for overcoming intrinsic and acquired resistance mechanisms in cancer therapy.

In summary, our results demonstrate that graphene oxide modulates the biological response of glioblastoma cells to bortezomib in a cell line-dependent manner. The combined treatment enhances oxidative stress and apoptotic signaling in selected GBM models; however, the underlying mechanisms remain incompletely understood. Future research should focus on refining nanocarrier systems, exploring combined treatment with agents that modulate autophagy or redox balance, and assessing responses in 3D spheroids, patient-derived GBM cells, glioma stem-like cells to better assess therapeutic efficacy, tumor microenvironment interactions, and clinical translational potential. Future studies addressing GO–drug interactions, ROS- and ER stress-dependent signaling, and validation in advanced preclinical models will be required before clinical translation can be considered.

## Data Availability

The data presented in this study are available on request from the corresponding author.

## Abbreviations

The following abbreviations are used in this manuscript:

GBM	Glioblastoma multiforme
BORT	bortezomib
ROS	reactive oxygen species
UPR	unfolded protein response
GO	Graphene oxide
MTT	3-(4,5-dimethylthiazol-2-yl)-2,5-diphenyltetrazolium bromide
EDTA	ethylenediaminetetraacetic acid
EGTA	ethylene-bis(oxyethylenenitrilo)tetraacetic acid
AMC	7-amino-4-methylcoumarin
